# State of Post-injury First Response Systems in Nepal—A Nationwide Survey

**DOI:** 10.3389/fpubh.2021.607127

**Published:** 2021-04-20

**Authors:** Amrit Banstola, Gary Smart, Raju Raut, Krishna Prasad Ghimire, Puspa Raj Pant, Prerita Joshi, Sunil Kumar Joshi, Julie Mytton

**Affiliations:** ^1^Faculty of Health and Applied Sciences, University of the West of England, Bristol, United Kingdom; ^2^Nepal Red Cross Society, Kathmandu, Nepal; ^3^Nepal Injury Research Centre, Kathmandu Medical College Public Limited, Kathmandu, Nepal; ^4^Department of Community Medicine, Kathmandu Medical College Public Limited, Kathmandu, Nepal

**Keywords:** emergency medical services, first aid, Nepal, organisation and administration, wounds and injuries

## Abstract

Injuries account for 9.2% of all deaths and 9.9% of the total disability-adjusted life years in Nepal. To date, there has not been a systematic assessment of the status of first response systems in Nepal. An online survey was cascaded through government, non-governmental organisations and academic networks to identify first response providers across Nepal. Identified organisations were invited to complete a questionnaire to explore the services, personnel, equipment, and resources in these organisations, their first aid training activities and whether the organisation evaluated their first response services and training. Of 28 organisations identified, 17 (61%) completed the questionnaire. The range of services offered varied considerably; 15 (88.2%) provided first aid training, 9 (52.9%) provided treatment at the scene and 5 (29.4%) provided full emergency medical services with assessment, treatment and transport to a health facility. Only 8 (47.1%) of providers had an ambulance, with 6 (35.3%) offering transportation without an ambulance. Of 13 first aid training providers, 7 (53.8%) evaluated skill retention and 6 (46.2%) assessed health outcomes of patients. The length of a training course varied from 1 to 16 days and costs from US$4.0 to 430.0 per participant. There was a variation among training providers in who they train, how they train, and whether they evaluate that training. No standardisation existed for either first aid training or provision of care at the scene of an injury. This survey suggests that coordination and leadership will be required to develop an effective first response system across the country.

## Introduction

Many Low- and Middle-Income Countries (LMICs) have inadequate pre-hospital trauma care (1–3), despite such countries carrying the greatest proportion of global trauma events. First response at the scene of an injury event is vital to improve outcomes, particularly in settings where Emergency Medical Services (EMS) are absent or limited (1). Improving access to high-quality first response care to victims of injury followed by good medical treatment has the potential to save lives, reduce injury severity and avert disability (4). Despite the importance of first response in reducing morbidity and mortality, it is only relatively recently that there has been evidence on such systems published from LMICs (5–8).

The World Health Assembly has urged member states to assess comprehensively their pre-hospital care context and includes it in emergency response plans (9, 10). In Nepal, there has not been a systematic assessment of first response systems, that has explored treatment providers, training organisations and the training levels provided. There is also a lack of information on outcomes, including the effects of any first responder interventions. Despite being predictable and largely preventable, injuries form an important public health problem in Nepal. The Global Burden of Diseases (2017) study estimated that injuries account for 9.2% (95% UI 7.5–11.3%) of all deaths and 9.9% (8.1–11.9%) of the total disability-adjusted life years in Nepal (11). This study aimed to investigate the current state of the first response system for trauma in Nepal, by identifying organisations providing the first response and assessing the distribution, activities and performance of these organisations.

## Methods

### Design of the Questionnaire

Two online questionnaires were developed specifically for this study using Qualtrics XM (Qualtrics, Provo, Utah, USA). The first questionnaire was designed to capture information about organisations providing first response. A first response provider was defined as an organisation that was involved in designing, implementing and evaluating first response and/or first aid programmes for road traffic crashes, natural disasters and other emergencies causing injuries. The second questionnaire sought to identify the services, personnel, equipment, and resources in these organisations, and their first response training activities. This survey included questions on the cost of first response training and whether the organisation evaluated the effectiveness of their training. Information about the study and a mandatory question to indicate the respondent gave consent to participate were included before the survey questions. The final questionnaire contained 68 items and was translated into Nepali.

### Validation of the Questionnaire

The questionnaires were reviewed for content validity by the members of a First Response Reference Group (FRRG); a stakeholder reference group of the Nepal Injury Research Centre (NIRC) consisting of 14 experts and key stakeholders involved in pre-hospital emergency medical, first response and first aid services in Nepal. The questionnaires were then pilot-tested with two organisations; the Nepal Red Cross Society and Good Neighbours International Nepal (formerly, Medical Teams International), and amendments were made before distribution.

### Participants Recruitment and Data Collection

The initial online survey was sent to FRRG members in December 2018. FRRG members were asked to cascade the survey through their networks and respondents were encouraged to snowball the survey onwards. First responder organisations identified in the first stage were provided with information on the study and invited to participate and complete the second questionnaire online, by phone or face-to-face. This second stage of data collection took place between December 2018 and December 2019. Organisations that were identified in the first stage but did not respond to the request to complete the main questionnaire online, were offered the opportunity to complete the questionnaire by phone or face-to-face survey. If after five emails and two phone calls no response had been obtained, they were considered non-participants and no further reminders were sent.

### Data Analysis

A de-identified dataset was exported from Qualtrics to Microsoft Excel format for analysis. Data were analysed descriptively to explore the provision, capacity and training reported by first response organisations in a Microsoft Excel 2019 Version 16.0 (Microsoft Corporation, Redmond, Washington, USA).

### Ethical Approval

Ethical approval for this study was obtained from the ethical review board of the Nepal Health Research Council (Reg. No. 551/2018) and the Faculty Research Ethics Committees of the University of the West of England, UK (Ref No. HAS.18.10.039).

## Results

Twenty-eight organisations were identified that provided post-injury first response services in Nepal. Of these, 17 completed the questionnaire (response rate 61%). Organisations which did not respond were more likely to be smaller, or national/international organisations that provide temporary emergency response to major disasters (such as earthquakes or floods). Responding organisations started their services between 1980 and 2018. The largest group of providers were Non-Governmental Organisations 10 (58.8%) (Table 1). First response organisations were available in all seven provinces and covered all 77 districts of Nepal, though province 3 (particularly Kathmandu District) was best served, with 13 (76.5%) providers providing a service in this district. Province 6 in western Nepal, appeared least well-served with only 5 (29.4%) service providers. Organisations varied in the services they provided; 15 (88.2%) provided first response training, 9 (52.9%) provided treatment at the scene, 8 (47.1%) provided ambulance facility, 6 (35.3%) provided transportation of the injured without an ambulance, and 5 (29.4%) provided full emergency medical services with assessment, treatment and transport to a health facility. First response training and the ambulance service were available in every district.

**Table 1:** General characteristics of organisations providing post-injury first response services in Nepal (*n* = 17).

**General characteristics**	**Number (%)**
**Organisation type**	
Public Sector Organisation	4 (23.5%)
Non-Governmental Organisation	10 (58.8%)
International Non-Governmental Organisation	1 (5.9%)
Intergovernmental Organisation	1 (5.9%)
Voluntary Group	1 (5.9%)
**Province**	
Province 1	6 (35.3%)
Province 2	6 (35.3%)
Province 3/Bagmati Pradesh	17 (100.0%)
Province 4/Gandaki Pradesh	10 (58.8%)
Province 5/Lumbini Pradesh	6 (35.3%)
Province 6/Karnali Pradesh	5 (29.4%)
Province 7/Sudurpashchim Pradesh	6 (35.3%)
**First response services**	
Ambulance service provider	8 (47.1%)
Any first response training provider	15 (88.2%)
Basic first aid training provider	13 (76.5%)
Advanced first aid training provider	6 (35.3%)
First aid Training of Trainers provider	6 (35.3%)
Transportation of the injured (without an ambulance)	6 (35.3%)
Emergency Medical Services	5 (29.4%)
Provide treatment at the scene	9 (52.9%)
**Injury specific training**	
Road traffic injury	5 (29.4%)
Natural Disasters	8 (47.1%)
Injuries (e.g., fall, burn, snakebites, poisoning)	4 (23.5%)
**Districts**	
Arghakhanchi, Baglung, Bardiya, Bhojpur, Dailekh, Darchula, Dhankuta, Dolpa, Doti, Eastern Rukum, Gulmi, Humla, Ilam, Jajarkot, Kalikot, Kapilvastu, Lamjung, Manang, Mugu, Mustang, Palpa, Panchthar, Parbat, Rolpa, Salyan, Sankhuwasabha, Syangja, Tanahun, Taplejung, Terhathum, Udayapur, Western Rukum	3 (17.6%)
Achham, Baitadi, Bajhang, Bajura, Bara, Dadeldhura, Dang Deukhuri, Jhapa, Jumla, Kanchanpur, Khotang, Myagdi, Nawalpur, Parasi, Pyuthan, Rautahat, Saptari, Sarlahi, Siraha, Surkhet	4 (23.5%)
Banke, Dhanusa, Kailali, Mahottari, Makwanpur, Okhaldhunga, Parsa, Rupandehi, Sindhuli, Solukhumbu, Sunsari	5 (29.4%)
Dhading, Kaski, Morang, Nuwakot, Rasuwa	6 (35.3%)
Dolakha, Gorkha, Ramechhap	7 (41.2%)
Chitwan	8 (47.1%)
Kavrepalanchok, Sindhupalchok	9 (52.9%)
Bhaktapur, Lalitpur	10 (58.8%)
Kathmandu	13 (76.5%)

### Ambulance Facility

A total of 308 ambulances for post-injury response were provided by 8 (47.1%) organisations participating in this study (Table 2). A driver, trained in first aid was available in 293 (95.1%) of ambulances.

**Table 2:** Ambulance services for post-injury.

	**Number (%)**
**Ambulance service stations**	
Kathmandu Valley	6 (26.1%)
Outside Kathmandu Valley	17 (73.9%)
**Number of ambulances**	
Kathmandu Valley	12 (3.9%)
Outside Kathmandu Valley	296 (96.1%)
**First aid trained ambulance driver**	
Yes	293 (95.1%)
No	15 (4.9%)
**Type of first aid training received by ambulance driver**	
Basic first aid training	291 (99.3%)
Basic life support	2 (0.7%)

### First Response Training

First response training included basic and advanced first aid and the training of first aid trainers. Training duration, training hours and the average training cost per participants differed according to the types of training (basic, advanced, training of trainers) and the participants (general population, students, health personnel, police, ambulance driver, community-based group) (Table 3).

**Table 3:** First response training types, duration, participants, and costs.

	**Training duration** ** (days)**	**Training hours** ** (per day)**	**Training participants** ** (in last 12 months)**	**Average training** ** cost per** ** participant (US$)**
	**Median** ** (Min, Max)**	**Median** ** (Min, Max)**	**Median** ** (Min, Max)**	**Mean** ** (Min, Max)**
**Basic first aid training for:**				
General population (*n* = 7)	3.0 (1.0, 4.0)	7.0 (3.0, 8.0)	175.0 (30.0, 1,752.0)	45.7 (0.0, 156.7)
Students (*n* = 3)	2.0 (1.0, 6.0)	8.0 (6.0, 8.0)	150.0 (50.0, 744.0)	14.5 (0.0, 30.5)
Health personnel (*n* = 7)	3.0 (1.0, 4.0)	7.0 (4.0, 8.0)	100.0 (25.0, 500.0)	46.9 (4.4, 87.0)
Police (*n* = 3)	3.0 (1.0, 4.0)	7.0 (6.0, 8.0)	15.0 (1.0, 144.0)	60.9 (52.2, 74.0)
Ambulance personnel (*n* = 5)	2.0 (1.0, 4.0)	7.0 (3.0, 8.0)	6.0 (4.0, 97.0)	35.9 (0.0, 74.0)
Community group (*n* = 5)	3.0 (1.0, 4.0)	8.0 (7.0, 8.0)	400.0 (100.0, 504.0)	59.2 (0.0, 156.7)
**Advanced first aid training for:**				
General population (*n* = 2)	7.5 (2.0, 13.0)	8.0 (8.0, 8.0)	2,144.0 (288.0, 4,000.0)	217.6 (130.6, 304.6)
Students (*n* = 1)	2.0 (2.0, 2.0)	8.0 (8.0, 8.0)	600.0 (600.0, 600.0)	13.1 (13.1, 13.1)
Health personnel (*n* = 3)	2.0 (2.0, 3.0)	8.0 (8.0, 8.0)	10.0 (1.0, 200.0)	162.5 (87.0, 304.6)
Police (*n* = 1)	2.0 (2.0, 2.0)	8.0 (8.0, 8.0)	200.0 (200.0, 200.0)	130.6 (130.6, 130.6)
Ambulance personnel (*n* = 2)	2.5 (2.0, 3.0)	8.0 (8.0, 8.0)	100.5 (1.0, 200.0)	265.5 (95.7, 435.2)
Community group (*n* = 2)	4.5 (2.0, 7.0)	8.0 (8.0, 8.0)	162.0 (24.0, 300.0)	326.4 (217.6, 435.2)
**First aid training of trainers for:**				
General population (*n* = 3)	7.0 (5.0, 9.0)	8.0 (8.0, 8.0)	72.0 (24.0, 150.0)	348.2 (156.7, 522.2)
Health personnel (*n* = 2)	5.0 (3.0, 7.0)	8.0 (8.0, 8.0)	2.5 (1.0, 4.0)	483.1 (130.6, 835.6)
Community group (*n* = 2)	4.0 (3.0, 5.0)	8.0 (8.0, 8.0)	15.5 (1.0, 30.0)	426.5 (156.7, 696.3)
Others (Teachers) (*n* = 1)	14.0 (14.0, 14.0)	8.0 (8.0, 8.0)	160.0 (160.0, 160.0)	391.7 (391.7, 391.7)

*Min, minimum; Max, maximum*.

*1US$ = NRs. 114.9 (as of 25 February 2020)*.

#### Basic First Aid Training

Basic first aid training lasted between 1 and 16 days, with 3–15 h of training per day. The most common participant was the general population and the training cost incurred by the participants ranged from US$4.4 to 156.7 per participant.

#### Advanced First Aid Training

Advanced first aid training lasted between 2 and 13 days with 8 h of training per day. The most common participant was the general population and the training cost incurred by the participants ranged from US$13.1 to 435.2 per participant.

#### First Aid Training of Trainers

Training of first aid trainers took between 3 and 14 days, with 8 h of training per day. The most common participant was a teacher and the training cost incurred by the participants ranged from US$130.6 to 835.6 per participant.

### Additional First Aid Training Related Information

Nine (69.2%) organisations who provided first aid training had their own trainers (Table 4). Basic first aid training equipment was not available in all organisations and not all organisations provided first aid equipment during the training exercises to their trainees. Eleven (84.6%) organisations reported that they have developed a first aid training curriculum or manual. Only 7 (53.8%) organisations evaluated skill retention of the trainees and only 6 (46.2%) collected health outcomes of patients receiving the first response, though all expressed willingness to collect these data in the future.

**Table 4:** Information provided by organisations providing first aid training (*n* = 13).

	**Number (%)**
**Who conducts the first aid/first response training?**	
Trainers provided by responding organisation	9 (69.2%)
Trainers hired from another organisation	5 (38.5%)
Foreign volunteers	2 (15.4%)
**How many trainers does the organisation have? (*****n*** **=** **9)**	
Median (IQR)	10 (6–20)
**What first aid (training) equipment for trauma is available with the organisation?**	
Resuscitation mannequins	9 (69.2%)
Wound dressings	11 (84.6%)
Burns dressings	8 (61.5%)
Cervical collars	9 (69.2%)
Splints	11 (84.6%)
Triangular bandages	9 (69.2%)
Chest seals	5 (38.5%)
Tourniquets	10 (76.9%)
Stretchers	10 (76.9%)
Automatic external defibrillators	5 (38.5%)
**What first aid equipment for trauma is provided during the training exercises to people who are trained by the organisation?**	
Wound dressings	6 (46.2%)
Burns dressings	3 (23.1%)
Cervical collars	4 (30.8%)
Splints	6 (46.2%)
Triangular bandages	5 (38.5%)
Chest seals	4 (30.8%)
Tourniquets	4 (30.8%)
Stretchers	5 (38.5%)
Automatic external defibrillators	2 (15.4%)
**Has the organisation developed a curriculum/guidelines/manual/ protocol as a first aid/first response training resource?**	
Yes	11 (84.6%)
No	2 (15.4%)
**Did the organisation follow-up trainees to assess their retention of skills?**	
Yes	7 (53.8%)
No	6 (46.2%)
**If the organisation follow up trainees, did the organisation have a report of follow up evaluation? (*****n*** **=** **7)**	
Yes	4 (57.1%)
No	3 (42.9%)
**If the organisation do not currently evaluate trainees skill retention was the organisation willing to commence collecting this information? (*****n*** **=** **6)**	
Yes	6 (100.0%)
**Has the organisation collected any information on the health outcomes of the patients receiving first response from the people who are trained by the organisation?**	
Yes	6 (46.2%)
No	7 (53.8%)
**If the organisation collect information on the health outcome of the patients receiving first response, did the organisation have a report of health outcome? (*****n*** **=** **6)**	
Yes	3 (50.0%)
No	3 (50.0%)
**If the organisation do not currently collect any information on the health outcomes of the patients receiving first response from the people who are trained by the organisation was the organisation willing to commence collecting this information? (*****n*** **=** **7)**	
Yes	6 (85.7%)
No	1 (14.3%)

*IQR, interquartile range*.

## Discussion

This nationwide survey is the first to provide a comprehensive account of the current state of post-injury first response systems in Nepal. Organisations that responded to our survey are the largest providers of first aid training and first response activities in the country. These organisations also participated in a national workshop on the standardisation of training programmes for emergency preparedness and response organised by the Government of Nepal and the World Health Organisation (WHO) Country Office Nepal (12), suggesting that they are influential in this field. Our findings show that there are multiple first response services in Nepal, with services provided by a range of different types of organisations (public, private, international or national non-governmental organisations, voluntary groups). All organisations appear independent and not coordinated. Despite involvement in a national workshop to standardise training for emergency preparedness, our survey indicated that there is no standardisation for community first aid training or for the routine provision of first response services.

### Ambulance Facility

Ambulance services for post-injury care were reported to be available across all provinces and districts of Nepal. The wide availability of ambulances across Nepal is because a small number of organisations such as the Nepal Red Cross Society operate in all districts of the country and can provide ambulance services through their district branches. It is important to note that these ambulances are also called upon for other medical emergencies such as obstetrics and paediatric emergencies. The ambulances may therefore not be available when called upon for post-injury rescue and transport or the waiting time for an ambulance may be increased (13, 14).

Of the ambulances described in this study, only one out of 17 organisations provided Class A ambulances (i.e., equipped to provide Advanced Life Support) and this organisation operated mostly in urban areas such as Kathmandu Valley. Although the authors are aware of the availability of air ambulance services in Nepal that information was not provided by the organisations that took part in this study. However, it is important to note that air ambulance services are provided by the modern tertiary and private hospitals using a helicopter, cover fixed geographical areas and are comparatively expensive (15–17).

Contrary to other studies from Nepal (13, 18), this study found that most 293 (95.1%) ambulances drivers were trained in basic first aid. Very few 9 (2.9%) ambulances were reported to have trained paramedics or Emergency Medical Technicians (EMTs). National guidelines on ambulance requirements state that ambulance drivers should be trained in first aid and have a minimum of 5 years of driving experience (19).

### First Response Training

This study showed variation among first aid training providers in who they train, how they train, and whether they evaluate that training. As is common in LMICs with limited EMS (20), in this study first aid training was reported to be provided to a wide range of people including the general population, students, health personnel, police, ambulance and commercial drivers, community groups, and teachers. The World Health Organization recommends the development of layperson first-responder programs as an essential step in establishing pre-hospital systems of care (3) and they have been shown to reduce the severity, disability and mortality resulting from injury (21–26).

This study found that only 6 (46.2%) out of 13 organisations providing training collected health outcomes from the patients who received first aid, though all expressed willingness to commence collecting these data in the future. In addition to implementing basic pre-hospital care systems, documenting each episode of pre-hospital care is vital for auditing mechanisms of injury, the treatment administered, and equipment used and then linking this through to the health outcomes (2).

### Emergency Medical Services

Five (29.4%) organisations that took part in this study reported they provide pre-hospital EMS. Pre-hospital EMS is well-established in most high-income countries but the provision is limited in LMICs (27–29). The Government of Nepal has a plan to improve EMS as part of a wider strategy to improve trauma care capacity in hospitals near major highways and major urban centres by 2021 (30). For this new system to work effectively, these trauma care facilities should be coordinated and provide a base for pre-hospital emergency medical services.

### Strengths and Limitations

This is the first study to examine the current status of first response system in Nepal. The study has explored the services, training providers and organisations including the availability of equipment and resources in these organisations to provide first response for road crashes, natural disasters and other emergencies causing injuries. The researchers sought support from their FRRG to identify organisations providing first response training in Nepal. Although a reliable source of knowledge, it is possible that smaller training organisations may have been missed, particularly in rural areas outside Kathmandu. The extent of such under-reporting is difficult to estimate, however, any effect has been mitigated by the use of “snowball” sampling and comparing the information against districts and provinces. Responding organisations may have been subject to social desirability bias, revealing selected or limited information. To minimise this risk, documentation to support their responses to the questionnaire was requested.

## Conclusions

There are multiple first response services in Nepal, however, they are independent and not coordinated. There was a variation among training providers in who they train, how they train, and whether they evaluate that training. No standardisation existed for either first aid training or provision of care at the scene of an injury. This survey suggests that coordination between first response service providers and the government will be required to develop common core training standards and an effective first response system across the country.

## Data Availability Statement

The original contributions presented in the study are included in the article/supplementary materials, further inquiries can be directed to the corresponding author/s.

## Author Contributions

AB, GS, SJ, and JM contributed to the conception and design of the study. AB and GS drafted the protocol design, methods, and data analysis plan. RR, KG, PP, and PJ supported the data collection. AB led the analysis, interpretation of data, and drafted the manuscript. GS, RR, KG, PP, PJ, SJ, and JM revised the manuscript critically for important intellectual content. All authors have approved the final version.

## Conflict of Interest

The authors declare that the research was conducted in the absence of any commercial or financial relationships that could be construed as a potential conflict of interest.
